# The Netrin-1/DCC guidance system: dopamine pathway maturation and psychiatric disorders emerging in adolescence

**DOI:** 10.1038/s41380-019-0561-7

**Published:** 2019-10-28

**Authors:** Daniel E. Vosberg, Marco Leyton, Cecilia Flores

**Affiliations:** 1 Department of Psychiatry, McGill University, Montreal, QC Canada; 2 Integrated Program in Neuroscience (IPN), McGill University, Montreal, QC Canada; 3 Neurology and Neurosurgery, McGill University, Montreal, QC Canada; 4 Douglas Mental Health University Institute, Montreal, QC Canada; 5 Present Address: Population Neuroscience and Developmental Neuroimaging, Bloorview Research Institute, Holland Bloorview Kids Rehabilitation Hospital, Toronto, ON Canada

**Keywords:** Addiction, Genetics, Neuroscience, Schizophrenia, Depression

## Abstract

Axon guidance molecules direct growing axons toward their targets, assembling the intricate wiring of the nervous system. One of these molecules, Netrin-1, and its receptor, DCC (deleted in colorectal cancer), has profound effects, in laboratory animals, on the adolescent expansion of mesocorticolimbic pathways, particularly dopamine. Now, a rapidly growing literature suggests that (1) these same alterations could occur in humans, and (2) genetic variants in *Netrin-1* and *DCC* are associated with depression, schizophrenia, and substance use. Together, these findings provide compelling evidence that *Netrin-1* and *DCC* influence mesocorticolimbic-related psychopathological states that emerge during adolescence.

## Objectives

The human brain is interconnected by an estimated 10 million km of neurites [1]. The specific routes taken are choreographed by a surprisingly small number of axon guidance molecules [2]. In this review, we summarize evidence that recently identified mutations and common variants of genes encoding the guidance cue Netrin-1 and its receptor, DCC (deleted in colorectal cancer), affect the adolescent expansion of mesocorticolimbic dopamine pathways and vulnerability to putative mesocorticolimbic-related psychiatric disorders.

## Mesocorticolimbic dopamine anatomy and psychiatric disorders

The primate mesocorticolimbic dopamine system shares many features with the homologous pathways in rodents. As in rodents, primate dopamine cells project from the upper brainstem to the dorsal striatum and multiple cortical and subcortical limbic regions [3, 4]. These latter targets include the ventral striatum (nucleus accumbens (NAcc), olfactory tubercle), septum, hippocampus, amygdala, and cortical regions, particularly the prefrontal (PFC), cingulate, and perirhinal cortices. Primates and rodents both have descending glutamatergic and GABAergic projections from the anterior cingulate and orbital frontal cortices to several limbic and midbrain regions, including the ventral striatum and the dopamine cell body regions, the substantia nigra (SN) and ventral tegmental area (VTA) [3, 5]. In both rodents and primates, the density of mesocortical dopamine fibers increases dramatically from adolescence to adulthood [6, 7]. This process, at least in rodents, results from dopamine axons continuing to grow beyond the NAcc to the PFC across adolescence [8]. Compared to rodents, primate cortical dopamine projections are more widespread [9], innervating the entire cortical mantle, albeit more to anterior than posterior regions [10, 11]. Subcortical dopamine axons are often myelinated in primates, a feature not seen in rodents [10].

**Fig. 1:**
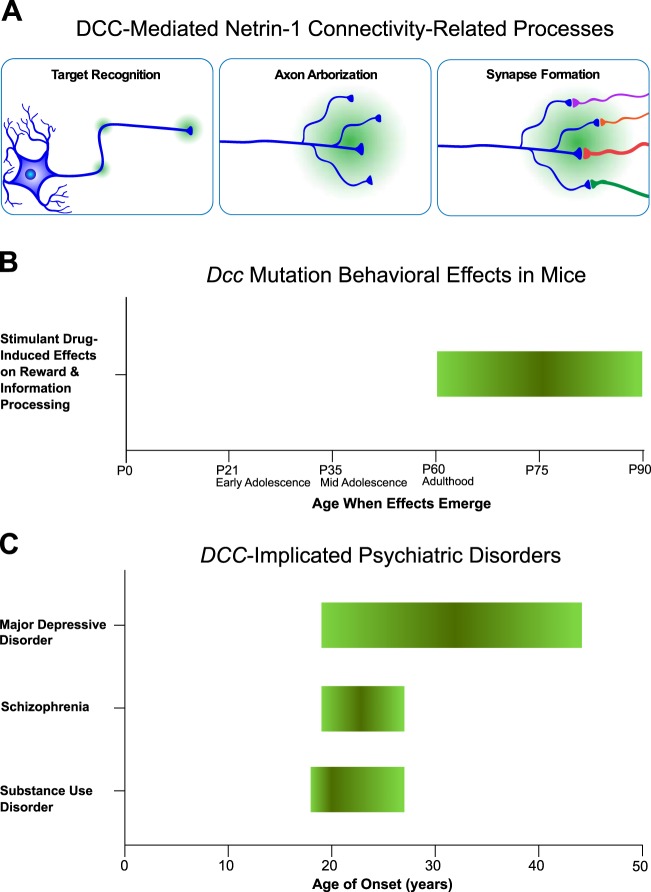
**a** DCC-mediated Netrin-1 connectivity-related processes. Throughout adolescent brain development, target recognition, axon arborization, and synapse formation are ongoing including dopamine axon targettting, long distance axonal growth, and synaptogenesis by mesocorticolimbic dopamine axons. Green gradients indicate Netrin-1 and the depicted axons  express DCC. **b** *Dcc* Mutation Behavioral Effects in Mice. Ages, in post-natal days (P), and periods (early adolescence, mid adolescence, and adulthood) at which stimulant drug-induced effects on reward and information processing emerge in *Dcc* haploinsufficient mice. The effects are in green, based on studies of *Dcc* haploinsufficient mice [6, 7]. **c** Age of Onset of *DCC*-implicated Psychiatric Disorders. The *DCC*-implicated psychiatric disorders, major depressive disorder, schizophrenia, and substance use disorder, begin to emerge in adolescence. The interquartile ranges (25th to 75th percentiles) are indicated in green. The median ages of onset for major depressive disorder, schizophrenia, and substance use disorder are 32, 23, and 20, respectively. Data to construct the figure were obtained from a U.S. survey and an international review [17, 18]

Disturbances to mesocorticolimbic development have been proposed to contribute to multiple psychiatric disorders. Consistent with this hypothesis, dopamine neurotransmission and mesocorticolimbic functional connectivity (the degree to which functional magnetic resonance imaging (fMRI) blood oxygen level-dependent (BOLD) signals from disparate brain regions are temporally correlated) are altered in the mesocorticolimbic system in schizophrenia [12], stimulant drug addiction [13, 14], and depression [15, 16], each of which begins to emerge in adolescence [17, 18]. The proposal here is that DCC-mediated Netrin-1 signaling alterations might be an important contributing factor [6, 7, 19] (Fig. 1).

## Axon guidance

The axon guidance properties of Netrin-1 and its receptor, DCC, are shared by a larger class of proteins which operate according to the following general mechanisms. During neurodevelopment, axon navigation is directed by extracellular axon guidance cues, which attract or repel growing axons by inducing molecular changes in their growth cones [2]. Growth cones are versatile structures with actin-based finger-like extensions (filopodia) and protruding sheets (lamellipodia) in their peripheral domains and microtubules in their central domains. Guidance cues induce elongation, retraction, or turning of growth cones, by altering the relative rates of polymerization and depolymerization in actin filaments as well as changes in microtubule stabilization of the growth cone’s cytoskeletal proteins, including actin filaments and microtubules [20]. These processes play a critical role in the organization of brain connectivity and are coordinated by a small number of guidance cue families, including the netrins, slits, semaphorins, and ephrins [2, 21]. There are many members of these families as well as splice variants [2, 21]. While the present review focusses on Netrin-1 and its receptor, DCC, there are additional Netrin-1 receptors including Neogenin [22], uncoordinated 5 homologues (UNC5-H) [23], and Down syndrome cell adhesion molecule (DSCAM) that contribute to the orchestration of neuronal networks [24]. Furthermore, in addition to guidance cue systems, cell adhesion molecules, such as neural cell adhesion molecules (NCAM) [25] and cadhedrins [26], as well as growth factors, also play critical roles in brain connectivity processes (i.e., synapse formation [27], axon branching [28], and axon guidance [29]).

## Netrin-1 and its DCC receptor

Netrin-1 is a mammalian laminin-related diffusible molecule that interacts attractively or repulsively with several receptors, including DCC [30]. These effects of Netrin-1 play a well-documented role in axonal pathfinding, an evolutionarily conserved process demonstrated in several species, including Drosophila [31], C. elegans [32], and rodents [6]. The role of netrin and its receptors continues beyond the laying down of pathway connectivity. Indeed, once axons reach their intermediate or final targets, Netrin-1 participates in connectivity-related processes, including target recognition, axon branching, synaptogenesis, and synaptic plasticity [11, 30, 32, 33].

DCC receptors are part of the immunoglobulin superfamily and their extracellular domains are composed of four immunoglobulin domains, in addition to six fibronectin type III repeats [34]. The fourth, fifth, and sixth fibronectin type III repeats constitute the binding sites for Netrin-1 [35]. In humans and rodents, the *DCC* gene is located on chromosome 18 and comprises 29 exons [36]. The first demonstration of DCC’s role in axon guidance was in commissural fibers of the developing neural tube [37], but DCC’s role is not restricted to these fibers or to this early stage. Instead, DCC remains expressed across the lifespan throughout the nervous system [38–40]. DCC and Netrin-1 are highly expressed in dopamine cell bodies and terminal regions, including the SN, VTA, striatum, hippocampus, and cerebral cortex, in both rodents and humans [38–45].

Mice bred for *Dcc* haploinsufficiency (+/−) have altered adolescent development of mesocorticolimbic dopamine neurons affecting dopamine transmission and dopamine-related behaviors in adulthood [6, 7]. Adult *Dcc*^+/−^ mice exhibit increased dopamine axon innervation, dopamine presynaptic sites, and amphetamine-induced dopamine release in the PFC. In comparison, in the NAcc, there are decreases in dopamine varicosities and amphetamine-induced dopamine release [8, 42, 45, 46]. The latter effects result from ectopic growth of mesolimbic dopamine axons to the PFC, a concomitant increase in mesocortical dopamine synapses [8], function [47], and augmented cortical inhibitory control over the responsiveness of mesolimbic dopamine neurons [48]. These changes are concordant with the findings that, as adults, but not as adolescents, *Dcc*^+/−^ mice display multiple alterations to dopamine-related behaviors, including diminished sensitivity to the effects of stimulant drugs (cocaine, amphetamine, methamphetamine) on locomotor activity, sensorimotor gating, conditioned place preference, and intracranial self-stimulation [6, 42, 49, 50]. These behavioral effects have been observed primarily under pharmacological challenge conditions but drug-free adult DCC-deficient mice exhibit reduced impulsivity [8] and attend less to a novel object in the presence of a familiar object [51]. These altered drug responses might be specific to stimulants, given that no differences in conditioned place preference responses are observed to either morphine or ethanol between *Dcc* haploinsufficient mice and controls (Personal Communication; Flores, Keifer, Darq and Nouel).

Behavioral and neurochemical effects of *DCC* haploinsufficiency are mirrored by *Netrin-1* haploinsufficiency. In adulthood, but not in adolescence, *Netrin-1* haploinsufficient mice exhibit increased medial PFC (mPFC) dopamine concentrations and reduced sensitivity to the behavioral effects of amphetamine [48]. Finally, adolescent amphetamine administration alters the expression of both DCC in dopamine neurons and Netrin-1 in the NAcc and mPFC [52].

In the following sections, we collate the evidence that similar DCC and Netrin-1 related effects occur in humans, influencing susceptibility to mood disorders, psychosis, and addictions. As described above, specifically during adolescence, DCC and Netrin-1 mediate dopamine axon targeting in rodents. These processes coincide with changing levels of a microRNA which suppresses DCC expression, miR-218 [7]. In comparison, directly testing temporal effects is generally not possible in human genetic association studies, given that the polymorphisms or mutations are present throughout the lifespan. Nonetheless, as depicted in Fig. 1, the observation that the psychiatric disorders associated with *DCC* and *Netrin-1* polymorphisms begin to emerge in adolescence raises the tantalizing possibility that the processes follow a parallel neurodevelopmental pathway.

## Human genetic investigations of *DCC*

### Schizophrenia

Several studies have linked *DCC* polymorphisms with schizophrenia. In a candidate gene study, comprising 556 schizophrenia patients and 208 healthy controls, a SNP (rs2270954) in *DCC* was found to be nominally associated with schizophrenia. It was postulated that because this SNP is within the regulatory 3′ untranslated region (UTR), it may alter *DCC* mRNA stability and consequently levels of DCC protein translation [53]. Given that the 3′UTR region contains microRNA (miRNA) binding sequences, the identified SNP may disrupt miRNA binding, potentially increasing DCC expression by preventing *DCC* mRNA transcript degradation and/or translation inhibition [54, 55].

A second candidate gene study of 454 patients with schizophrenia and 486 healthy controls reported a nominal association with a *DCC* SNP (rs2229080) on exon 3 [56]. The authors also found evidence that rs2229080 induces a protein structural change and, according to in silico analyses, alters splicing regulation. Moreover, the authors noted that rs2229080 is a known target of loss of heterozygosity (LOH) and that such LOH is associated with reduced DCC expression [57]. Thus, if the rs2229080 risk allele disrupts the LOH target site (which downregulates DCC), the authors proposed that the risk allele would result in increased DCC expression, changing mesocorticolimbic dopamine development and ultimately contributing to the schizophrenia phenotype [56]. Subsequently, a much larger genome wide association study (GWAS), applying a false discovery rate (FDR) correction, found that an intronic locus of *DCC* (rs4632195) is associated with schizophrenia (*n* = 82,315) [58]. A mechanism regarding predicted expression outcomes remains to be determined for this SNP. Most recently, by applying next-generation sequencing, which sequences the entire genome and can detect rare variants, loci in five genes, including *DCC*, were shared among three family members exhibiting atypical psychosis [59].

### Depression

Over the past six years, there has been rapidly accumulating evidence that both genetic variants and other factors that alter *DCC* expression also affect susceptibility to mood dysregulation and suicide. Two independent studies from our group, in a discovery and replication cohort, have demonstrated that depressed suicide completers exhibit elevated *DCC* mRNA expression in the PFC, and a corresponding downregulation of the *DCC* miRNA repressor, miR-218 [54, 60]. Moreover, a genome-wide investigation of differential gene expression in blood, applying a Bayesian approach, identified 165 differentially expressed genes in major depressive disorder, including overexpression of *DCC* [61]. Further strengthening these findings is a blood-derived methylome-wide association study (MWAS) of 812 patients with depression and 320 controls, which found associations between methylation sites in *DCC* and depression [62]. Notably, while there is evidence of general concordance between DNA methylation across blood and brain tissue, there are exceptions [63], and this is a limitation of the study.

GWAS research has identified an intronic *DCC* SNP (rs4542757) associated with depressive symptoms (*n* = 3138) [64]. Although this effect did not achieve genome-wide significance and was not identified in a replication sample, the GWAS study was likely underpowered [64]. A larger GWAS study (*n* = 161,460) identified an association between depressive symptoms and an intronic *DCC* SNP, rs62100776 [65]. Moreover, using pathway analyses in two independent samples (*n* = 6455, *n* = 18,759), FDR-corrected associations between depression and a Netrin-1 signaling pathway were identified, comprising SNPs from multiple genes involved in Netrin-1 signaling, including *DCC* [66]. Additionally, gene-based tests found that depressive symptoms among participants in the UK Biobank (*n* = 99,057) were associated with six genes, including *DCC* [67]. Another UK Biobank (*n* = 122,935) gene-based analysis found associations between suicidality and five genes, including *DCC* [68]. Finally, a GWAS meta-analysis of 135,458 individuals with major depression and 344,901 controls identified 44 genomic loci significantly associated with depression, including an intronic *DCC* SNP (rs11663393) [69].

The above associations might reflect an effect on mood instability, a clinical feature common to numerous psychiatric disorders [70]. In a GWAS study of 60,443 controls and 53,525 mood instability cases, genome-wide significance was detected for four independent genetic loci, including an intronic *DCC* SNP (rs8084280) [71]. In line with this idea, genetic correlations, which assess the degree of shared heritability between phenotypes, were identified between mood instability and three psychiatric conditions: major depressive disorder, schizophrenia, and anxiety disorder [71].

Recently, a UK Biobank study (*n* = ~6400) found that the SNPs in the Netrin-1 signaling pathway conferring risk for major depression are associated with altered white matter microstructure in thalamic radiations, namely lower fractional anisotropy and higher mean diffusivity [72].

Strikingly, a UK Biobank meta-GWAS (*n* = 375,275) identified an association between anhedonia and a locus in *DCC*, which was the most statistically significant finding [73]. The authors also reported high genetic correlations between anhedonia and depression, as well as a moderate genetic correlation with schizophrenia [73]. Moreover, a higher anhedonia polygenic score predicted reduced brain volumes, including in the NAcc and mPFC, as well as altered white matter integrity in multiple pathways [73].

Furthermore, a genome-wide methylation study of 150 pairs of monozygotic twins (one co-twin with, and one without, early onset major depression), identified altered methylation in *Netrin-1*, among other genes in depression [74]. An additional meta-GWAS study reported that a *Netrin-1* SNP, rs8081460, was associated with neuroticism (which is highly genetically correlated with depression) in the UK Biobank sample (*n* = 91,370), although this SNP effect did not replicate in two smaller, independent samples (*n* = 6659 and *n* = 8687) [75].

The larger number of studies implicating DCC, relative to Netrin-1, variants is notable. We propose that changes in receptor expression/function, including DCC, result in modifications in Netrin-1′s actions (attracting or repelling). Therefore, subtle spatiotemporal variation in DCC expression could be sufficient to produce changes in connectivity, even if total Netrin-1 expression is unaltered [6].

In the largest cross-disorder meta-GWAS of neuropsychiatric disorders to date, comprising more than 232,964 cases and 494,162 controls across eight disorders, the intronic *DCC* SNP, rs8084351, had the most robust pleiotropic effects [76]. This striking finding indicates that the effects of *DCC* and *Netrin-1* are important across a wide variety of psychiatric disorders.

### *DCC* haploinsufficiency: personality traits and drug-related behaviors

While GWAS studies typically detect relatively subtle effects of *DCC* polymorphisms [77], loss-of-function haploinsufficient *DCC* mutation carriers were expected to exhibit larger effects, detectable with smaller sample sizes. Our group recently conducted neuroimaging and psychological studies of a large Quebec family (*n* = 36), half of whom possess a heterozygous frameshift mutation to *DCC* (NM_005215.3, c.1140 + 1 G > A). The resulting mutated allele encodes a truncated DCC protein that fails to bind to Netrin-1 [78]. As in *Dcc* haploinsufficient mice, robust anatomical and behavioral phenotypes are observed as a consequence of human *DCC* haploinsufficiency, underscoring the sensitivity of the system. The *DCC* haploinsufficient Quebecers have an adult behavioral phenotype that shares two striking features with adult *Dcc* haploinsufficient mice [79]. First, the adult *DCC* haploinsufficient humans exhibit reduced novelty seeking personality traits [79]. Second, compared with their unaffected relatives, the *DCC* haploinsufficient humans smoke less tobacco yet use similar amounts of alcohol and cannabis [79], consistent with the evidence in mice that DCC’s effects are specific to stimulant drugs. Notably, cigarette smoking increases dopamine transmission in humans [80] while lowered dopaminergic tone can decrease smoking [81], indicating that an altered smoking phenotype could reflect alterations to the dopamine system.

These findings are bolstered by a large meta-GWAS (*n* = 518,633), which identified associations between an intronic *DCC* SNP, rs1221976, and self-reported “ever smoker” [82]. This finding was part of a larger study on risk-tolerance, whereby *DCC* SNPs were also associated with “adventurousness”, defined as the propensity to be “adventurous versus cautious.” An additional UK Biobank GWAS study (*n* ~ 458,000) identified an intronic *DCC* SNP, rs12970816, associated with cigarette smoking status [83]. Finally, one more study (discovery: *n* = 5339, replication: *n* = 1682) reported that the intronic *DCC* SNP, rs1372626, while not genome-wide significant, was the SNP most strongly associated with cigarette smoking, and was plausibly underpowered [84].

Moreover, in a human multivariate investigation, using a powerful and sensitive alternative to traditional SNP studies, *DCC* was among the top genes associated with impulsivity (*n* = 426) [85]. Since diminished novelty seeking is associated with reduced dopamine release in the ventral striatum of humans and rodents [14, 86, 87] these behavioral alterations might reflect DCC’s effects on mesocorticolimbic dopamine development and striatal dopamine transmission [42].

### *DCC* haploinsufficiency: dopamine mesocorticolimbic connectivity

The associations between *DCC* and psychiatric disorders might be a consequence of *DCC*-related alterations to mesocorticolimbic pathways. As predicted, our group revealed that *DCC* haploinsufficient members of the Quebec family, as compared with control groups without the mutation (i.e., both relatives and unrelated healthy volunteers) exhibit striking reductions in anatomical connectivity, assessed using diffusion MRI probabilistic tractography, from the SN/VTA to both the ventral striatum and ventral mPFC [79].

These effects might include changes to dopamine pathways, but some caution is warranted. First, the reduced mesocortical connectivity differs from the increased cortical dopamine innervation seen in adult *Dcc* haploinsufficient mice. Second, MRI methodologies do not discern the underlying neurochemistry. Indeed, since mesocorticolimbic pathways contain dopamine, gamma-aminobutyric acid (GABA) and glutamate axons [88], the anatomical connectivity findings in humans plausibly represent alterations to both dopaminergic and non-dopaminergic axons. These same considerations also raise the possibility that the dopamine focused studies in rodents have yet to identify alterations to inter-connected non-dopamine neurons.

In rodents, there is a complementary receptor to ligand expression pattern of DCC and Netrin-1 in the NAcc and PFC. While dopamine axons express high DCC levels in the NAcc, they only rarely express DCC in the PFC [45]. Conversely, the intensity of Netrin-1 expression in the NAcc is low, especially compared to the PFC, where Netrin-1 expression is substantial [45]. Indeed, dopamine axons expressing high levels of DCC target the NAcc and do not continue to grow to the PFC in adolescence [8]. In the case of *Dcc* haploinsufficiency, since DCC expression is reduced, mesolimbic dopamine axons fail to recognize the NAcc as their final target and instead continue to grow ectopically into the PFC throughout adolescence [8]. In human *DCC* mutation carriers with reduced mesocortical anatomical connectivity, these same effects might be occurring yet the larger distances to be covered might lead the misrouted mesocortical axons to disperse more diffusely, compared to the rodents.

### Striatal brain volume

We recently reported that both *DCC* haploinsufficient humans and mice exhibit reduced striatal volumes. While these effects occur in the NAcc in mice, they are localized to the putamen in humans [79]. These effects were also identified in large-scale GWAS investigations. One of these GWAS studies (*n* = 30,717), part of the Enhancing Neuro Imaging Genetics through Meta-Analysis (ENIGMA) initiative, investigated genetic variants associated with the volumes of subcortical structures [89]. Putamen volume was associated with four genetic loci, including an intronic *DCC* SNP (rs62097986), in both discovery and replication cohorts [89].

Bilateral putamen volume has also been associated with an intronic SNP in *DCC* (rs62098013), as identified in the UK Biobank Brain Imaging Data browser (http://big.stats.ox.ac.uk/), which comprises neuroimaging GWAS data from 9,707 participants [90]. Confidence in this finding is bolstered by a more recent GWAS study that reported an additional intronic locus of *DCC* (rs4632195) associated with both putamen volume (*n* = 11,598) and schizophrenia (*n* = 82,315) [58]. The risk allele for schizophrenia is associated with larger putamen volumes.

Earlier work also identified larger putamen volumes among those with schizophrenia [91], consistent with the two genetic investigations that found associations between SNPs in *DCC* and schizophrenia [53, 56]. This observation fits well with our earlier proposal that schizophrenia is associated with increased *DCC* expression [53]. Moreover, in discovery (*n* = 905) and replication (*n* = 166) cohorts, variants in genes incurring risk for schizophrenia, including *DCC*, were associated with alterations in gray matter volumes (putamen, thalamus, temporal gyrus), resting state functional magnetic resonance imaging (rs-fMRI) signals in the mPFC, and working-memory performance [92].

These striatal volumetric effects might have implications for mood disorders as well. In depression, there are reports of decreased putamen volume [93–95], although some other groups have failed to replicate this finding [96] potentially reflecting small sample sizes, heterogeneity within the diagnostic category, and medication effects. Indeed, there is recent MRI evidence that, in psychotropic medication-naïve participants (*n* = 625), elevated putamen gray matter volume is a disease risk marker across multiple diagnostic categories, namely schizophrenia, major depression, obsessive compulsive disorder, and post-traumatic stress disorder [97].

### Cortical volume

The *DCC* haploinsufficient Quebecers also demonstrate modest volumetric increases in two cortical regions: the mPFC/anterior cingulate cortex and the ventral mPFC [79]. In comparison, among human carriers of another *DCC* mutation, a completely different phenotype is observed, such that there is a complete absence of the cingulate gyrus [98]. Volumetric cortical changes are not observed in *Dcc* haploinsufficient mice [79] and have not been identified in human neuroimaging GWAS investigations, to the authors’ knowledge, and therefore, the cortical findings may have limited generalizability.

## Identification of different *DCC* SNPs

**Fig. 2:**
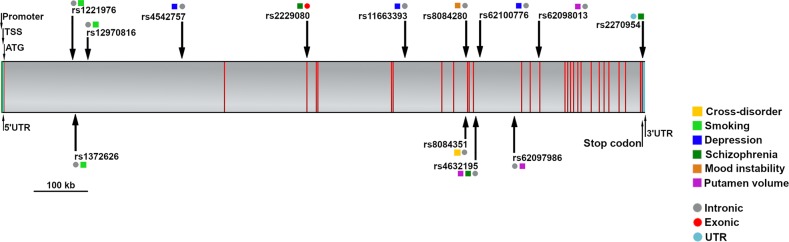
Single nucleotide polymorphisms (SNPs) and associated phenotypes in the *DCC* gene. Depicted here is the 5′−3′ oriented *DCC* gene, comprising 29 exons (red), intervening introns (gray), untranslated regions (UTR; teal), transcriptional start site (TSS) region, promoter region, start codon (ATG) and stop codon. The rs ID for each SNP and associated phenotype(s) are indicated. The phenotypes are cross disorder (yellow), smoking (light green), depression (blue), schizophrenia (dark green), mood instability (orange), and putamen volume (purple). The gene structure and SNP locations were determined using the NCBI tool, Variation Viewer (https://www.ncbi.nlm.nih.gov/variation/view/), using the genome assembly, GRCh38.p12

The effects reported here are related to multiple SNPs (Fig. 2). This could reflect several factors, including linkage disequilibrium, the set of SNPs examined, the genetic compositions of samples, the statistical procedures, the sample sizes (i.e., statistical power), and the differing use of covariates. Of note, 85% of the discussed *DCC* SNPs are intronic, indicating that they plausibly influence splicing, and consequently, *DCC* mRNA transcription and translation; [99] such intronic SNPs may affect enhancers or repressors which may distally regulate *DCC* transcription [100].

## Conclusions

This review highlights accumulating evidence that Netrin-1 and its receptor DCC contribute to mesocorticolimbic dopamine related psychiatric disorders that emerge during adolescence (Tables 1 and 2). The findings are strikingly convergent across heterogenous methodologies and samples and both between and within studies in humans and mice.

**Table 1:** Summary of human genetic studies of *DCC* and *Netrin-1*

References	Genetic approach	Gene	Sample size	Ancestry	Phenotype
*Mood disorders*					
Manitt et al. [60]	mRNA expression	*DCC mRNA*	30 cases, 35 controls	Not available	Depressed suicide completers
Dunn et al. [64]	Genome-wide association study (GWAS)	*DCC* gene	3138	Hispanic	Depression
Okbay et al. [65]	GWAS	*DCC* gene	161,460	European	Depression
Smith et al. [75]	Meta-GWAS	*Netrin-1*	91,370, replication samples: (6659, 8687)	White, United Kingdom	Neuroticism
Torres-Berrío et al. [54]	mRNA expression	*DCC mRNA*	11 cases, 12 controls	Not available	Depressed suicide completers
Zeng et al. [66]	Pathway analysis, multilevel regional heritability, and polygenic risk score	Netrin-1 signaling pathway	25,214	European	Depression
Aberg et al. [62]	Methylome-wide association study (MWAS)	*DCC* methylation sites	812 cases, 320 controls	European	Depression
Leday et al. [61]	Genome-wide gene expression	*DCC mRNA*	207 cases, 157 controls	Caucasion	Depression
Roberson-Nay et al. [74]	Genome-wide methylation study	*Netrin-1*	150 monozygotic twin pairs	Caucasion	Depression
Wray et al. [69]	GWAS	*DCC* gene	135,458 cases, 344,901 controls	European	Depression
Arnau-Soler et al. [67]	Gene-based test	*DCC* gene	99,057	White, United Kingdom	Depression
Barbu et al. [72]	Polygenic risk score	Netrin-1 signaling pathway	~6400	Not available	Depression
Lee et al. [76]	Meta-GWAS	*DCC* gene	232,964 cases, 494,162 controls	European	Cross-disorder
Strawbridge et al. [69]	Gene-based test	*DCC* gene	122,935	White, United Kingdom	Suicidality
Ward et al. [73]	Meta-GWAS, polygenic score, genetic correlations	*DCC* gene	375,275	European	Anhedonia
*Personality traits and substance use*					
Khadka et al. [85]	Parallel independent component analysis	*DCC* gene	426	Caucasion, African-American, Hispanic, other	Impulsivity
Zanetti et al. [84]	GWAS	*DCC* gene	5339, replication: 1662	African-American	Cigarette smoking
Ward et al. [71]	GWAS	*DCC* gene	53,525 cases, 60,443 controls	White, United Kingdom	Mood Instability
Vosberg et al. [79]	Rare mutation cohort	*DCC* gene	20 cases, 36 controls	Caucasion French Canadian (cases)	Novelty seeking & tobacco use
Kichaev et al. [83]	GWAS	*DCC* gene	*n* ~ 458,000	European	Cigarette smoking
Lee et al. [76]	Meta-GWAS	*DCC* gene	232,964 cases, 494,162 controls	European	Cross-disorder
Linnér et al. [82]	Meta-GWAS	*DCC* gene	*n* = 518,633	European	Cigarette smoking
*Schizophrenia and psychosis*					
Grant et al. [53]	Candidate gene	*DCC* gene	556 cases, 208 controls	African American, Asian, Caucasion	Schizophrenia
Yan et al. [56]	Candidate gene	*DCC* gene	454 cases, 486 controls	Han Chinese	Schizophrenia
Okayama et al. [59]	Next-generation sequencing	*DCC* gene	3 cases	Japanese	Atypical psychosis
Smeland et al. [58]	GWAS	*DCC* gene	82,315	European, East Asian	Schizophrenia
Lee et al. [76]	Meta-GWAS	*DCC* gene	232,964 cases, 494,162 controls	European	Cross-disorder
*Neurobiology*					
Hibar et al. [89]	GWAS	*DCC* gene	30,717	European	Putamen Volume
Elliot et al. [90]	GWAS	*DCC* gene	9707	White, United Kingdom	Putamen Volume
Luo et al. [92]	Polygenic risk score	Schizophrenia-associated genes including *DCC*	Discovery (*n* = 905); replication (*n* = 166)	Han Chinese	Putamen, thalamus, temporal gyrus volumes; mPFC rs-fMRI activity; working-memory
Smeland et al. [58]	GWAS	*DCC* gene	11,598	European	Putamen volume
Vosberg et al. [79]	Rare mutation cohort	*DCC* gene	20 cases, 36 controls	Caucasion French Canadian (cases)	Mesocorticolimbic anatomical connectivity & putamen volume
Barbu et al. [72]	Polygenic risk score	Netrin-1 signaling pathway	~6400	Not available	Thalamic raditions, white matter integrity

**Table 2:** Summary of DCC and Netrin-1 effects across brain regions

Brain region	Measure	Mice	Humans
Striatum	A. Expression	High DCC, low Netrin-1	Both Netrin-1 and DCC are expressed and their expression decreases across the lifespan
	B. Volume	Reduced in ventral striatum	Reduced in dorsal striatum
	C. Connectivity	Reduced mesolimbic dopamine innervation	Reduced mesolimbic anatomical connectivity
Cortex	A. Expression	Low DCC, high Netrin-1 (mPFC)	Higher DCC than Netrin-1 (prenatally)
	B. Volume	No difference	Reduced
	C. Connectivity	Increased mesocortical dopamine innervation	Reduced mesocortical anatomical connectivity

(A) DCC and Netrin-1 relative expression levels in wild-type mice [7] and humans [39] (See also: http://development.psychencode.org). Group differences in (B) volumes and (C) connectivity in *DCC* haploinsufficient mice [7] and humans [79], relative to control groups
